# A Residual Fusion Network for Osteosarcoma MRI Image Segmentation in Developing Countries

**DOI:** 10.1155/2022/7285600

**Published:** 2022-08-03

**Authors:** Jia Wu, Luting Zhou, Fangfang Gou, Yanlin Tan

**Affiliations:** ^1^School of Computer Science and Engineering, Central South University, Chang Sha 410083, China; ^2^Research Center for Artificial Intelligence, Monash University, Clayton Vic 3800, Melbourne, Australia; ^3^PET-CT Center, The Second Xiangya Hospital of Central South University, Changsha 410083, China

## Abstract

Among primary bone cancers, osteosarcoma is the most common, peaking between the ages of a child's rapid bone growth and adolescence. The diagnosis of osteosarcoma requires observing the radiological appearance of the infected bones. A common approach is MRI, but the manual diagnosis of MRI images is prone to observer bias and inaccuracy and is rather time consuming. The MRI images of osteosarcoma contain semantic messages in several different resolutions, which are often ignored by current segmentation techniques, leading to low generalizability and accuracy. In the meantime, the boundaries between osteosarcoma and bones or other tissues are sometimes too ambiguous to separate, making it a challenging job for inexperienced doctors to draw a line between them. In this paper, we propose using a multiscale residual fusion network to handle the MRI images. We placed a novel subnetwork after the encoders to exchange information between the feature maps of different resolutions, to fuse the information they contain. The outputs are then directed to both the decoders and a shape flow block, used for improving the spatial accuracy of the segmentation map. We tested over 80,000 osteosarcoma MRI images from the PET-CT center of a well-known hospital in China. Our approach can significantly improve the effectiveness of the semantic segmentation of osteosarcoma images. Our method has higher F1, DSC, and IOU compared with other models while maintaining the number of parameters and FLOPS.

## 1. Introduction

Osteosarcoma derives from the primitive bone-forming mesenchymal cells and is the most common primitive bone malignancy [1]. The first peak of osteosarcoma is during puberty and the second is after 65 years old when it affects adults. The deaths caused by osteosarcoma take 8.9% of all cancer-related deaths in children and adolescents. If not diagnosed timely, the 5-year survival rate of patients with detectable metastases at the time of diagnosis has the reports as low as 19%. In comparison, the survival rates of patients with pulmonary and bony metastases are 0% at four years, which can be considered deadly [2]. As a study shows, with the current status of medical conditions, there will be 11.1 million children dying of cancer, 84.1% of whom come from low or lower-middle-income countries [3]. The cancer diagnosis and treatment in developing countries are far behind those in developed countries, with low automation applied. Therefore, the timely diagnosis of osteosarcoma for developing countries is significant and needs to be improved.

The diagnosis of osteosarcoma is mainly judged by the radiographic appearance of the affected bones. Cross-sectional imaging techniques such as CT scans or MRI can represent the extent of osteosarcoma invasion of bone and soft tissue vividly [4]. However, CT is less often used to scan and detect the primary tumors, since MRI can better visualize statuses like soft tissue extension, localized intramedullary metastases, and intramedullary beating metastases [5]. Therefore, doctors often make use of MRI images to provide a thorough evaluation and diagnosis of patients.

However, we can only rely on doctors to do it manually when we want to give a diagnosis and detect the tumor area, and the low doctor-patient ratio in developing countries increases the difficulty to provide a timely targeted diagnosis for every patient [6, 7]. Each patient produces over 600 MRI images during a single diagnosis, but often less than 20 of which can contribute to the final decision. The massive redundant data result in doctors spending prolonged time judging the validity of the produced images. Besides, the position, size, and structure of osteosarcoma vary from patient to patient, and sometimes the distinction between osteosarcoma tumors and other tissues is not apparent, requiring doctors to pay full attention during assessment [8–10]; the misdiagnosis rate of inexperienced doctors is high. Thus, the final result will require more experienced experts to perform further assessments to obtain. In developing countries, the scarcity of medical resources intensified this situation, and the low doctor-patient rate led to large amounts of patients hardly receiving a definitive diagnosis.

With the development of computer technology, some image processing approaches are continuously being applied in the clinical diagnosis of MRI images [11–13]. Computer-aided detection (CAD) systems are often used to assist physicians during the diagnosis process [14]. The systems are capable of image processing, possible lesion areas localizing, feature selection and extraction, as well as classification and segmentation. This has alleviated the difficulties in diagnosing osteosarcoma caused by the lack of medical resources in developing countries to some extent. In recent years, with the development of artificial intelligence and its effective application in many realms [15–17], CNN-based designs have gradually entered the limelight and are used broadly in image segmentation areas. Encoder-decoder-based CNNs allow for deep, semantically important, fully-connected feature maps to be passed from encoders to decoders due to their skip-connection features.

However, the optimal depth of such design is unknown, and their skip connections will lead to unnecessary restrictive fusion patterns [18–20]. Only equally scaled feature maps in the encoder and decoder subnets can be aggregated, and the processing of data in different dimensions is not precise and comprehensive [21]. In the meantime, a massive amount of data are often required when training an RNN with good results [22], and due to the expensive nature of MRI images, it is often difficult to get enough data for practical training.

To address these issues, we propose a CNN-based approach using the residual fusion network for osteosarcoma MRI image segmentation (RFNOMS). To alleviate the lack of data, we augment the original data by rotating, transposing, and flipping. The design includes BFE blocks used for binomial feature exchange and a subnetwork constructed with BFE blocks to achieve multiscale feature exchange. To address the problem of low accuracy when processing data in different dimensions, we use the BFE block to get inputs of two different dimensions and use a residual fully-connected block to exchange messages between them after each convolutional layer. The feature of this block allows for the complete transfer of both high-level and low-level features to the final prediction, enabling the production of more spatially accurate predictions. By making the redundant BFE blocks gradually decrease during the propagation, this multilayer residual network allows only the most valuable features, which are the ones that contribute most to the prediction of the segmentation mask. Due to the irregular boundary and complex shape of osteosarcoma tumors, we add a shape flow to the network to calculate the edges of the masks more accurately.

The main contributions of this paper are as follows:

1. RFNOMS computes multiscale features during data transfer, and then exchanges and fuses the messages through BFE blocks. The BFE block improves the gradient flow and thus increases training efficiency and allows for data exchange between features of different scales.
2. By using multiple BFE blocks for information exchange among various dimensions, the network enables successful transmission of both high- and low-resolution features in the osteosarcoma image, preserving the most semantically meaningful features through the process and producing more spatially accurate predictions.
3. By using channel and spatial attention, as well as gated attention mechanisms to acquire semantic information, we can identify the target regions of the osteosarcoma MRI images, as well as process the different kinds of semantic information separately to preserve the semantically meaningful ones.
4. The data set we use of more than 80,000 samples is collected from the MRI images of pancreatic cancer patients from the Second Xiangya Hospital of Central South University. These images contain a variety of standard MRI image classifications of osteosarcoma, and our network achieves better results than other SOTH methods on the data set. The method provides an automatic approach for the diagnosis and treatment of osteosarcoma, and the output images can assist the doctors in their identifying significantly, greatly reduce the amount of time required for judgment, release their stress and thus increase diagnostic accuracy.

The paper is arranged as follows: the second section introduces some research related to our work, the third section depicts the main structure and design of our network, and the fourth section shows the performance of the model through evaluation metrics and compares different aspects of the model with other models through similar experiments. At the end of the paper, we summarize our work and present possible future applications as well as improvements.

## 2. Related Work

Medical image segmentation includes inputting digital grey-scale images (i.e., CT or MRI) and outputting the predicted masks. The purpose is to extract information that can help with diagnoses from medical images, such as the possible position of the tumor, to reduce the amount of manual labor required and assist the physician in the diagnosis [23–25]. Various methods such as neural networks, decision trees, and Bayesian networks have been applied to receive the desired segmentation map output [26].

Pixel-based methods include thresholding, where pixels that meet a given criterion are considered part of the target [27], and clustering, which is done by dividing the images into multiple clusters [28]. Edge-based detection methods generally involve two steps: edge detection and concatenation, and active contour models are often used in the process. Among the region-based methods, most are based on the nature of regions and boundaries [29], the most common are region growth and region segmentation merging [30]. In the meantime, structure-based methods provide a better symbolic description of the image. In contrast, artificial neural network (ANN)-based methods do not rely on probability density distribution functions and considerably reduce the need for human intervention. Methods based on fuzzy set theory can analyze and provide accurate information for any image [31]. There are also methods based on genetic algorithms, which use natural evolution-like methods such as inheritance, natural selection, mutation, and hybridization to obtain the solutions [32].

With the increase in GPU performance and the consequent trend towards deep learning, there are also many deep learning techniques applied to medical image analyses, in particular convolutional network methods [33–35]. The best-known image processing method based on CNN is the U-Net, proposed by Ronneberger et al. They use the same number of upsampling and downsampling layers and connect between opposing convolution and deconvolution layers with skip connections. This enables features on the contracting and expanding to connect, allowing for processing the whole image during one forward propagation and generating a segmentation map. Zhan et al. [36] proposed a CNN-based intelligent medical system that assists the diagnosis processes, capable of semantic segmentation of nonsmall cell lung cancer. In addition to the ANN and CNN methods mentioned above, RNNs are also frequently applied in segmentation methods. Xie et al. [37] proposed a spatial clock RNN that uses the current prior information from the row and column predecessors of the current patch. Srivastava et al. [38] obtain a more accurate segmentation map through data exchanges between different resolution scales and improved the accuracy and DSC effectively. Chen et al. [39] combined a 2D fully-connected FCN network with a bidirectional LSTM-RNN to separately process intra- and intercontext, improving compatibility between highly anisotropic dimensions. Poudel et al. [40] added gated cyclic units to the 2D U-Net architecture for image segmentation, significantly reducing computation time while simplifying the segmentation process.

In recent years, some of the approaches mentioned above have been applied to the study and diagnosis of osteosarcoma. Glass et al. [41] proposed segmentation and classification method using dynamic contrast-enhanced MRI images. They were one of the pioneers to use a fully-automated method for the MRI image processing of osteosarcoma, eliminating intra- and interoperator mistakes caused by manual manipulation. Yu et al. [42] proposed a medical decision system for cancer treatment, aiming at the current medical situation in developing countries. Siddharth et al. also studied image segmentation using computational intelligence techniques [43–45] and applied computer vision-based approaches to leaf disease segmentation and classification [46, 46]. Mishra et al. [47] used the CNN architecture and improved the efficiency and accuracy of osteosarcoma classification. Their proposed architecture consists of 8 layers, with three sets of connected convolutional layers and max-pooling layers to extract the features and two fully-connected layers to enhance the results. Their method can effectively classify the images by whether it contains a tumor or not, but cannot localize the position specifically. Arunachalam et al. [48] combined the pixel and object-based methods to classify different regions of the tumor. They took into account several characteristics of osteosarcoma including nuclear cluster, density, and circularity, but their approach can only determine whether a given region is a tumor region and is unable to segment the image and find the boundary of the tumor.

In general, although many image processing methods using machine learning have been proposed, few have been applied to the semantic segmentation of osteosarcoma, and even fewer have optimizations that took into account the classification of osteosarcoma images. Therefore, to fill the gap in efficient and accurate methods that are used for semantic segmentation of osteosarcoma, we proposed a residual fusion network-based semantic segmentation method for MRI images of osteosarcoma. By comparing it with other commonly used semantic segmentation methods, this method can be targeted to improve efficiency and effectiveness and has good generalization.

## 3. System Model Design

Due to the extreme lack of medical resources in developing countries, most people in rural and urban areas are unable to find a doctor with sufficient credibility to make a diagnosis. For the multiple reasons discussed above, the cost of diagnosis and treatment is so high that most people will choose to save up the money for their lives over spending tons of money diagnosing a disease they might not have. Particularly, because osteosarcoma is common in the adolescent population, which is the age group that is often considered the least possible to be diagnosed with cancer and whose health issues are easily overlooked, it leads to fewer people being screened for this type of cancer. And like most malignancies, osteosarcoma is much easier to treat when diagnosed early, and the difficulty improves greatly as the tumor develops. These reasons can easily lead to diagnoses too late to treat and save lives.

**Figure 1:**
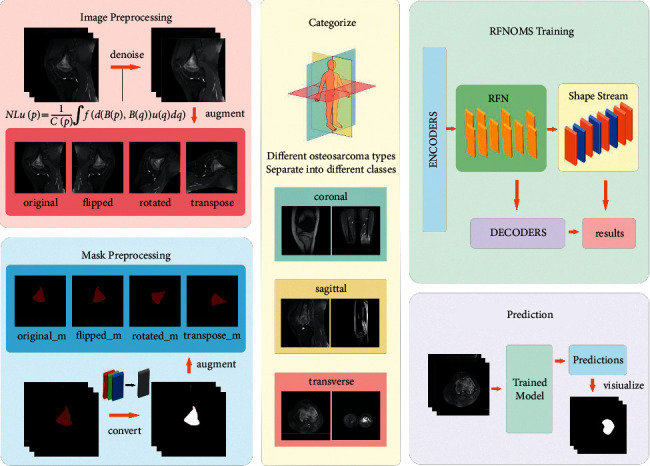
The main structure of our system.

Therefore, to reduce the tragedy of untimely diagnosis, we need to find ways to reduce the cost of osteosarcoma diagnosis as soon as possible and decrease the overall cost and increase accessibility, i.e., using machines instead of manual detection [49]. With the development of machine learning and computer-aided detection (CAD), more algorithms have been proposed and optimized [50–52], and more and more machine learning methods are being used to assist doctors in the diagnosis process. To effectively reduce the burden on physicians and substitute with machines, our approach provides efficient and accurate semantic segmentation of osteosarcoma MRI images to identify and display the most likely areas of morbidity. Based on this, our proposed RFNOMS is primarily used to assist physicians in identifying the location of osteosarcomas. The general structure of RFNOMS is shown in Figure 1. Some of the symbols and their paraphrases are listed in Table 1.

### 3.1. Data Preprocessing

Large data sets containing well-labeled medical images are difficult to obtain, especially those annotated by authoritative physicians. To obtain the best segmentation results, we should also use data sets with high-quality labels, so the standards of data needed for training are often difficult to satisfy, resulting in difficult data collection of osteosarcoma MRI images. In the meantime, digital preservation and exchange of medical images in developing countries are incomplete owing to a lack of supervision and communication, making it difficult to exchange data effectively between different hospitals.

Therefore, we need to use functional methods to augment the data set. In our experiments, we used three expansion methods: rotating the image 45° clockwise, transposing the image, and flipping the image. By using these common but effective extensions, we quadruple the input data, meeting the sufficient data volume required for training the model.

One of the most significant factors that affect the effectiveness of MRI image processing is the noise in the images. Gaussian noise is a type of noise generated during the acquisition of MRI images. Gaussian noise causes the images to be polluted in certain spots and therefore will negatively affect the segmentation process of them. To deal with this situation, we used the NLM noise reduction algorithm to reduce the noise, using the denoised images as the model input. At the same time, since the masks were stored in RGB mode while only containing data of one dimension, we transformed them into *L* mode to decrease the total data amount for the convenience of the reading and prediction process.

### 3.2. Network Design

**Figure 2:**
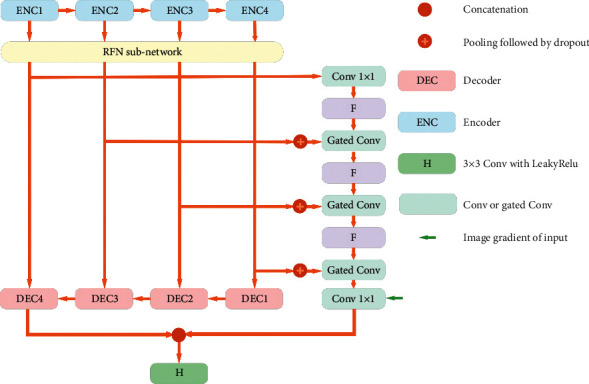
RFNOMS design.

The network design of RFNOMS is divided into four main sections: the encoder, the RFN, the shape flow block, and the decoder block. The general structure is shown in Figure 2.

#### 3.2.1. Encoders

There are four encoders in total, ENC1-4. Each encoder consists of two successive convolutional layers with succeeding squeeze and excitation (S and E) blocks. The S&E block improves the performance of the structure by enabling dynamic cross-channel feature correction to cover more locations where osteosarcoma may be present. To aggregate the feature maps in the spatial dimension of the channels, we first use global average pooling, then use the set of each channel weight in the excitation step to obtain cross-channel dependencies. For each encoder, we downscaled the resolution using a maximum pooling of Step 2 and normalized the model using drop out, where *p* for drop out is set to 0.2.

#### 3.2.2. BFE Block

**Figure 3:**
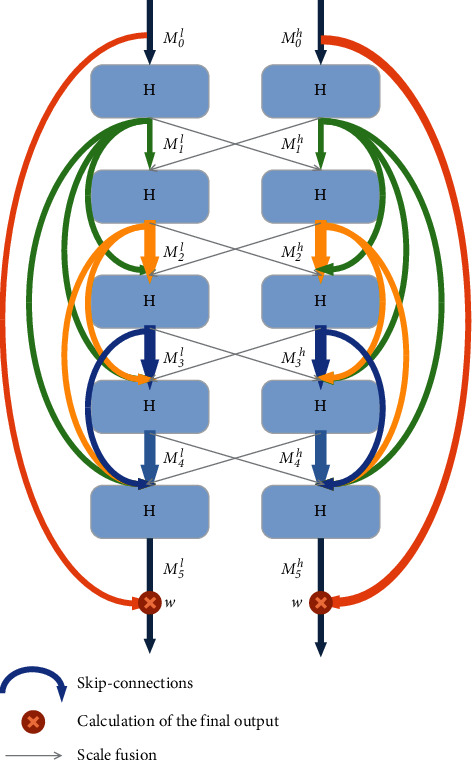
BFE block.

The three major types of osteosarcoma images include the sagittal plane, coronal plane, and transverse plane, each containing different resolutions of semantic information. Therefore, to make the target image semantically richer and more spatially accurate, we need to maintain the resolution of various osteosarcoma image features during feature encoding. The information exchange between different scales is achieved by BFE blocks. The BFE blocks are capable of preserving low-level features and optimizing the data flow, while the original resolution maintains.  Figure 3 depicts the architecture of the BFE blocks. The BFE blocks set up two parallel routes for different resolution scales, separating the high- and low-resolution aspects of an osteosarcoma MRI image for processing. Each propagation route is constructed of a fully-connected residual block and five *H* structures. The input of the nth *H* is *D*_*n*_^*s*^, where

(1) $$\begin{array}{c} s=\left\{\begin{array}{cc} l, & \text{low resoultion route}\\ h, & \text{high resoultion route}, \end{array}\right.. \end{array}$$

The formula can be represented as

(2) $$\begin{array}{c} M_{n}^{h}=H\left(M_{n-1}^{h}\cap T\left(M_{n-1}^{l}\right)\cap M_{n-2}^{h}\cap \cdots \cap M_{0}^{h}\right),\\ M_{n}^{l}=H\left(M_{n-1}^{l}\cap T\left(M_{n-1}^{h}\right)\cap M_{n-2}^{l}\cap \cdots \cap M_{0}^{l}\right), \end{array}$$

where ⌒ represents concatenation operation and *n* ∈ [1,5]and *M*_0_^*s*^ represents the initial output on the *s* route. The number of output channels of the *H* structure represents the growth factor, which is used to specify the number of new osteosarcoma features that can be extracted and propagated by a layer. As each scale has a different growth factor, we use only two scales at a time to make training more feasible and reduce the computational complexity of the model. Also, we used local residual learning to optimize the data flow and residual scaling to prevent instability that could lead to poor semantic segmentation results for osteosarcoma. The scaling parameter is factor *w* ∈ [0,1] and set to 0.4. The final output of the BFE block is

(3) $$\begin{array}{c} O_{s}=w\times M_{5}^{s}+M_{0}^{s}. \end{array}$$

#### 3.2.3. RFN

**Figure 4:**
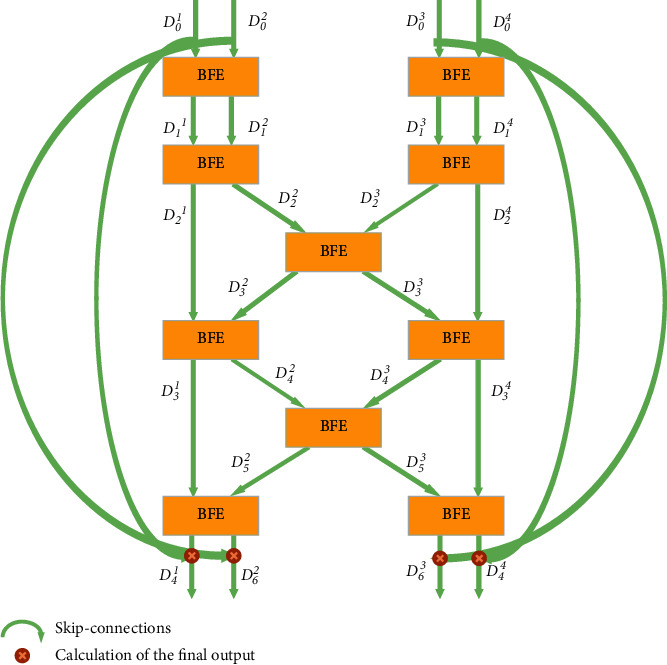
RFN subnetwork.

The RFN subnet includes multiple BFE blocks to exchange information between multiple dimensions in the osteosarcoma image. The structure is shown in Figure 4.

In algorithm 1, we define the input as dividing all pairs of resolution scales and placing them, respectively, into BFE blocks. Each layer includes four resolution scales. *A* and *B* represent high- and low-resolution sets of features, denoting each block by *a* and *b*. At the same time, the $\hat{D}$ represents the exchange of osteosarcoma image features in the central BFE. After the fourth RFN subnet layer, features can be exchanged efficiently, and global multiscale fusion is obtained. For any *r* ∈ [1,4] , *D*_0_^*r*^ can pass its osteosarcoma MRI image features through multiple BFE blocks to all the parallel resolution representations. Thus, we can exchange features between all scales efficiently with this approach. Therefore, we can handle the features of osteosarcoma images in different resolutions separately and fuse them. Similar to the BFE blocks, the first layer of the subnetwork outputs is added to the original RFN subnetwork input. This multilayer residual network can effectively preserve the most helpful features to osteosarcoma semantic segmentation and allows for the redundant features to die out through the BFE blocks.

#### 3.2.4. Shape Flow

The shape of osteosarcoma varies from person to person, and the boundaries can be ambiguous due to the blurred connection with bones and other tissues. We use gated shape flow to perform shape prediction to obtain more precise boundaries. The BFE block extracts relevant high-level feature representations that contain essential data, such as the outline of a specific osteosarcoma tumor, and passes them through the shape flow. Let  *p*  be the number of layers. We use bilinear interpolation to allow the spatial dimensions of *D* and *D*_0_^*r*^ to match, then the attention table for the gated convolution can be calculated as

(4) $$\begin{array}{c} \alpha_{p}=\sigma \left({\text{Conv}}_{1\times 1}\left(S_{p}\cap D\right)\right), \end{array}$$

where *σ*() represents the sigmoid activation function. The *D*_*p*+1_ is

(5) $$\begin{array}{c} D_{p+1}=F\left(S_{p}\times \alpha \right). \end{array}$$

We concatenate the output (depicted by × ) and gradients of the original input image and fuse it with the segmentation stream and feed it into the final *F* operation. Therefore, we could obtain more accurate predictions of the semantic segmentation of osteosarcoma by improving the spatial accuracy of the segmentation map.

#### 3.2.5. Decoders

**Figure 5:**
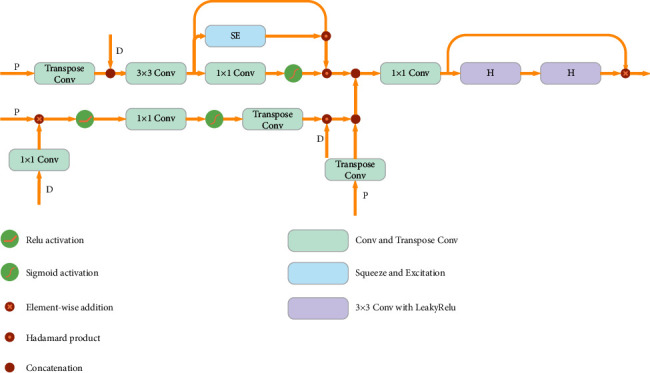
The structure of a decoder block.

In the decoder blocks, we use attention mechanisms, as shown in Figure 5. Two attention mechanisms are used in our structure. The first applies channel as well as spatial attention. The second involves an attention gating mechanism for acquiring contextual information *s* and identifying the osteosarcoma region and its structure. We used squeeze-excitation blocks to calculate cross-channel coefficients, denoted by SE. At the same time, we use a 1 × 1 convolutional layer to reduce the number of input channels from *n* to 1 for the same channel and calculate its spatial attention. After this, we use a sigmoid activation to scale the coefficients to produce activation maps and obtain the result *M* after superimposing *n* times. The spatial and channel attention output is expressed as

(6) $$\begin{array}{c} A_{\text{SC}}=\left(\text{SE}+1\right)⊙M, \end{array}$$

where ⊙ represents the Hadamard product and SE being the squeeze and excitation block. In the attention gating mechanism, the attention factor is calculated as

(7) $$\begin{array}{c} A_{AG}=T\left(\sigma \left({\text{Conv}}_{1\times 1}\left(\phi \left(F\right)+\phi \left(P\right)\right)\right)\right)⊙D, \end{array}$$

where *ϕ* represents a convolution function with a step size of 2 and a 1 × 1 kernel size with *n* channels of output, *φ* represents a convolution function with a step size of 1 and a 1 × 1 kernel size, *F* represents two *H* structures and a skip connection, and *P* is the output of the previous block.

Among the decoders, the input of DEC1 is the output of ENC4 after being processed by the RFN subnetwork, while the input for the other decoders is the skip-connection residual blocks and the output of the previous DEC block. The features from *ϕ* and *φ* are combined and put into a 1 × 1 convolutional layer to change the number of output channels to 1. Beyond this, a sigmoid activation function is used to obtain the activation map, and then a *T* operation is performed to obtain *A*_AG_. After that, we perform Hadamard multiplication along with *F* to remove the features that are irrelevant to the predicted osteosarcoma region, allowing for the features that contribute to osteosarcoma MRI image segmentation to be transmitted further. The update of *A*_AG_ is

(8) $$\begin{array}{c} A_{\mathrm{AG}}=A_{\mathrm{AG}}\cap T\left(P\right). \end{array}$$

We combine the channel attention, spatial attention, and gated attention to obtain the output of the decoder block:

(9) $$\begin{array}{c} A_{\mathrm{SCC}}=A_{\mathrm{SC}}\cap A_{\mathrm{AG}}. \end{array}$$

Then, it is input into two *H* structures that represent a consecutive 3 × 3 convolution layer with a LeakyRelu activation to create the final output. The output is then visualized as a mask and printed for the doctor to diagnose.

The proposed model can be used for real-time processing of MRI images by inputting the MRI images of the patients and outputting the possible location of the tumor as a mask. The model can provide an efficient and effective aid to doctors, improve their diagnostic efficiency, and reduce the rate of misdiagnosis caused by humans. It will significantly reduce labor costs and make the diagnosis more affordable and reliable. In clinical practices, the model can run on a wider range of devices as it is less demanding on hardware and can provide accurate masked predictions of the location of osteosarcoma, which is a great assistance to doctors in the diagnosing process of osteosarcoma.

## 4. Simulation Analysis

### 4.1. Data sets

The MRI image data set of osteosarcoma we trained with was provided by the Education Mobile Health Information Department—China Mobile Joint Laboratory and the Second Xiangya Hospital of Central South University [53]. We collected over 80,000 MRI images from 204 osteosarcoma patients from the PET-CT center of the hospital over time, and the ground truth maps are annotated by a group of experienced physicians. For practical use, we used a train/validation/test ratio of 7 : 2 : 1. The characteristics of patients are shown in Table 2.

### 4.2. Evaluation Metrics

We used a variety of standard medical image segmentation evaluation metrics, including intersection over union (IoU), dice similarity coefficient (DSC), sensitivity, accuracy, precision, recall, and F1-score. We used the true positive (TP), true negative (TN), false positive (FP), and false negative (FN) from the confusion matrix to obtain these indicators. The following are the calculations of the relevant indicators.

The intersection over union (IoU) is one of the most commonly used metrics for semantic segmentation and, as the name suggests, represents the ratio of the intersection over the union between the predicted semantics. We assume that *I*_1_ is the predicted tumor region and I_2_ is the actual region, then the IoU can be calculated by the formula as follows:

(10) $$\begin{array}{c} \text{IoU}=\frac{I_{1}\cap I_{2}}{I_{1}\cup I_{2}}. \end{array}$$

The dice similarity coefficient (DSC) is a validation index for reproducibility and a spatial overlap index [54]. It represents the ratio of the area of the intersection of the predicted doubled and actual regions to the sum of their areas. Accuracy represents the proportion of correct judgments, while precision and recall are used to judge the model's accuracy. The F1-score can be used to balance precision and reca ll, is a measurement of the model's accuracy, and reflects the overall validity of the model. As we are performing medical image processing, we aim to assist doctors in making diagnoses, improving accuracy, and reducing the rate of misdiagnosis. We, therefore, try to make DSC as high as possible during training to prevent misdiagnosis.

### 4.3. Comparison Algorithms

1. U-Net [55] allows for end-to-end training with very few images by using large amounts of data augmentation to make use of the available data more efficiently. The architecture includes a compressed path for acquiring information and an extended path for performing precise localization.
2. SepUNet [56] proposes a model that significantly reduces the number of parameters required while maintaining a high level of accuracy and can effectively reduce the computational cost associated with single-coil reconstruction tasks.
3. MSFCN [57] introduces a multisupervised side output layer in a deep end-to-end network to guide multiscale feature training. A large number of feature channels are used in upsampling to obtain more information to improve accuracy.
4. MSRN [58] adds three supervised side output modules to the residual network, among which the deep side output module can extract semantic features and the shallow output module can extract the shape features of images, and fusing the results of the three modules can calculate the final segmentation result.
5. FPN [59] uses the pyramidal hierarchy structure and the inherent multiscale of deep convolutional networks to construct a feature pyramid and built high-level semantic feature tables at all scales to perform semantic segmentation.

### 4.4. Training Methods

The model was run on an NVIDIA A40 GPU and implemented using Tensorflow. The residual network was trained for 200 epochs. The Adam optimizer was used, the learning rate was set to 0.0001, and the size of input images was 512 × 512.

### 4.5. Evaluation of Segmentation Results

**Figure 6:**
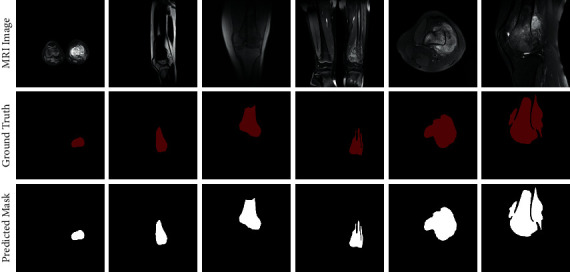
Results compared to ground truth.

The three rows in Figure 6 are the original MRI image, the ground truth, and the predicted mask. Red masks in the ground truth row are annotated by experienced doctors, and the white masks in the third row are the predicted results of the model. As the figure shows, the segmentation results indicate that the model performs extremely well, correctly segmenting tumors regardless of their location in the MRI image. At the same time, it can accurately depict the corresponding boundaries of osteosarcoma even when the shape of the osteosarcoma is complex. It is capable of handling the MRI images that are often similar to each other, assisting in the detection and diagnosis of osteosarcoma. The model provides compelling predictions for all types of osteosarcoma MRI images, with little difference from the ground truth masks. The results demonstrate the model's ability to process all types of osteosarcoma images.

**Figure 7:**
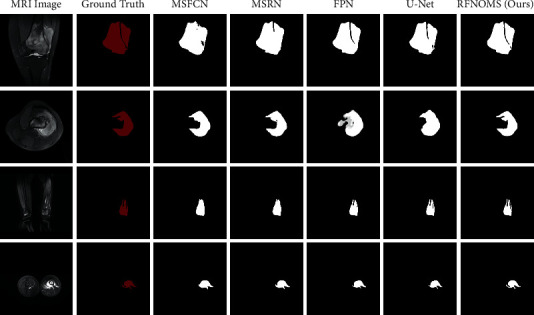
Comparison between different model predictions.

Figure 7 shows the segmentation results of different models. The first column shows the original input image, the second contains ground truth images, and the rest shows the segmentation results of several different models, with the final column showing the results of our structure. We can judge the effectiveness of the models based on the similarity to ground truth maps and compare the predictions. We can see that RFNOMS can segment the original image better and shows clear boundaries and good segmentation results. In particular, when it comes to the specific details of the mask such as tumors separated by bones or other irregular shapes, which is a common occasion, our model can segment the affected region clearly with clean boundaries and match the ground truth accurately. Whether sagittal, coronal, or transverse, the model can perform accurate semantic segmentation to obtain effective masks for all kinds of osteosarcoma MRI classes.

Table 3 compares the various types of values between the models.

This table shows that our model has higher precision than the other models, indicating that it can predict the tumor's location more accurately. The F1 is also the highest, meaning that the overall performance of precision and recall is better and can effectively perform segmentation. The DSC was significantly higher than the other models, reaching 0.929, showing the high repeatability of segmentation and high spatial overlap accuracy. In the meantime, the amount of parameters and the FLOPS of the architecture can be kept low, which means the prediction effect can be guaranteed without high hardware requirements, providing an effective solution for doctors in clinical environments. At the same time, our model can achieve an accuracy of more than 99.1% to obtain the possible location of the target region precisely.

**Figure 8:**
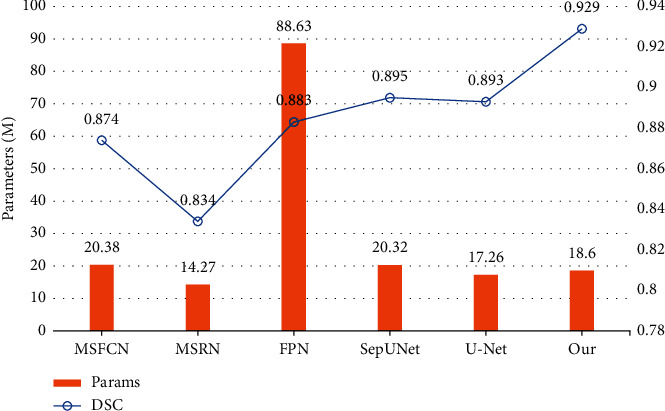
Comparison of parameter size and DSC between models.

As can be seen from Figure 8, our model has a high DSC with a low number of parameters, indicating that it can obtain good segmentation results with low hardware requirements and achieve an accuracy-efficiency trade-off. While improving the DSC up to 0.929, our model does not require a lot more parameters, with 18.60 M, which is below the average of all the models in comparison.

**Figure 9:**
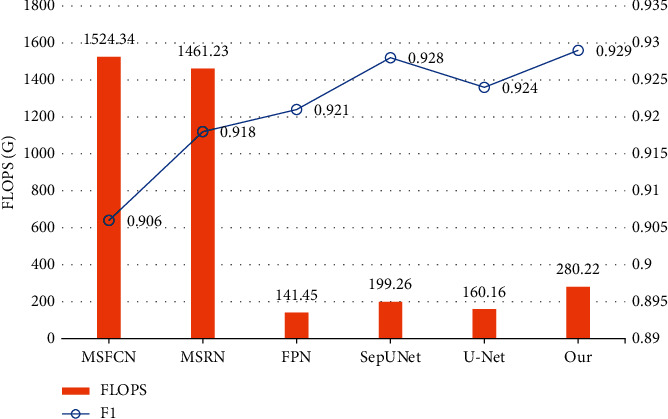
Comparison of FLOPS and F1-score between models.

As Figure 9 demonstrates, the FLOPS of our model keeps at a relatively low state, i.e., the segmentation can stay fast and therefore keep the waiting time short, providing a possible method for real-time clinical prediction. The F1-score gets a solid increase to 0.929, indicating we can give accurate predictions to targets effectively, producing more accurate predictions overall and assisting doctors in their medical diagnoses.

**Figure 10:**
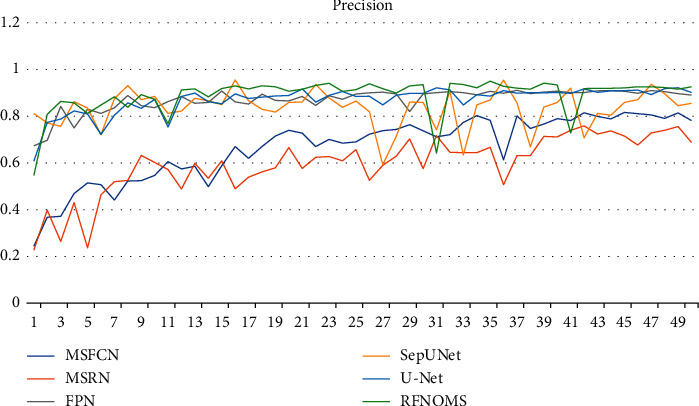
Variation of precision in different models' training process.

**Figure 11:**
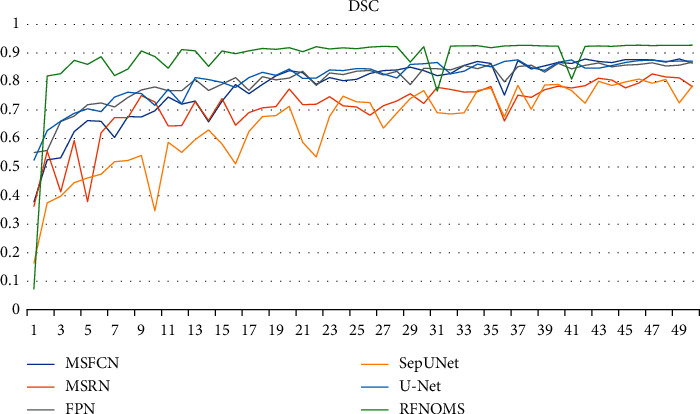
Variation of DSC in different models' training process.

**Figure 12:**
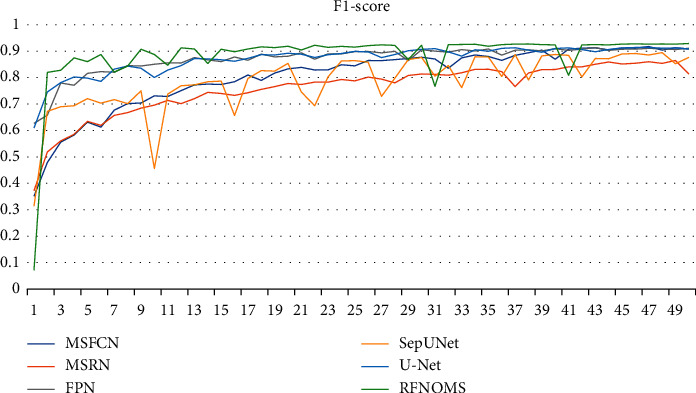
Variation of F1-score in different models' training process.

**Figure 13:**
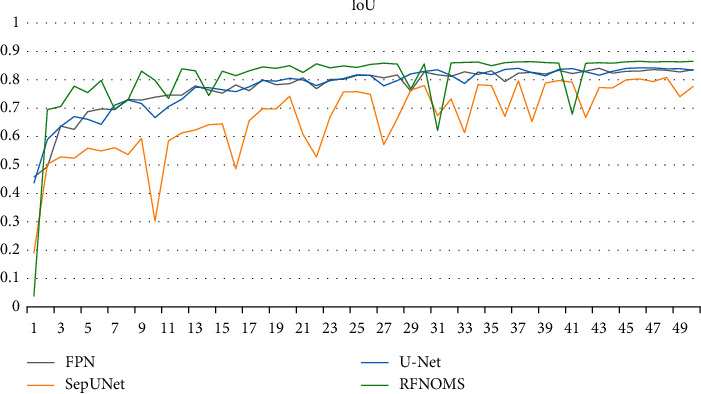
Variation of IoU in different models' training process.

Figures 10–13 show the variationof different evaluation metrics duringthe training process, thenumbers on the horizontal plane have been scaled from [1, 200] to [1, 49] to present the data more comfortably.

## 5. Conclusion

In this article, we performed semantic segmentation on MRI images of osteosarcoma using a residual fusion network, using data sets from osteosarcoma patients in three Chinese hospitals. Our approach fuses information between two different scales using BFE blocks and fuses information among multiple scales by combining multiple BFE blocks in the RFN subnet. At the same time, we incorporate gated shape flow for shape prediction and used a combination of channel attention, spatial attention, and gated attention mechanisms. The model can segment osteosarcoma images effectively with little computational cost increase, while the accuracy and efficiency of the segmentation process are improved, improving the overall assistance.

In our future research, we plan to introduce other improvement methods into the model, such as graph representation and point-voxel fusion. We will try to integrate more optimization methods and image preprocessing approaches to improve the prediction accuracy while preventing overfitting issues. We will continue to follow the research progress in computer vision and try to integrate more network structures and image representation methods to optimize the segmentation method when the edges of the tumor fuse with the tissue around. [4].

## Figures and Tables

**Algorithm 1:**
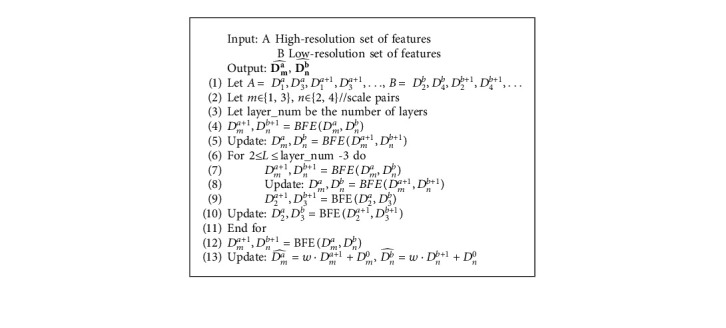
RFN subnetwork.

**Table 1:** Some of the symbols in this chapter.

Symbol	Paraphrase
*H*	3 × 3 convolution block with a LeakyRelu activation
*C*	Convolution block with 3 × 3 core and stride 2
*T*	Transpose convolution layer with 3 × 3 core and stride 2
*F*	Residual block with 2 *H* operations and a skip connection
SE	Squeeze and excitation block
*s*	Representation of h(high) in high-res routes and l(low)in low-res routes
*w*	The scaling factor
*D*_*n*_^*s*^	The input of the *n*th *H* structure
*M*_*n*_^*s*^	The output map of the *n*th *H* structure
$\hat{D_{m}^{a}}$ , $\hat{D_{n}^{b}}$	The output of the RFN subnetwork
*S*_*l*_	Shape stream feature map of the lth layer
*A*_*SC*_	The output of spatial and channel attention block
*A*_*AG*_	The output of the attention gate block
*P*	The output of the previous decoder block

**Table 2:** The baseline of patient characteristics.

Characteristics		Total *N* = 1144	Training set *N* = 800 (69.9%)	Validation set *N* = 229 (20.0%)	Test set *N* = 115 (10.1%)
Age	<15	269 (23.5%)	188 (23.5%)	54 (23.6%)	27 (23.5%)
	15–25	735 (64.2%)	515 (64.4%)	147 (64.2%)	73 (63.5%)
	>25	140 (12.3%)	97 (12.1%)	28 (12.2%)	15 (13.0%)

Gender	Female	516 (45.1%)	361 (45.1%)	103 (45.0%)	52 (45.2%)
	Male	628 (54.9%)	439 (54.9%)	126 (55.0%)	63 (54.8%)

Marital status	Married	179 (15.6%)	124 (15.5%)	36 (15.7%)	19 (16.5%)
	Unmarried	965 (84.4%)	676 (84.5%)	193 (84.3%)	96 (83.5%)

SES	Low SES	437 (38.1%)	306 (38.3%)	87 (38.0%)	44 (38.3%)
	High SES	707 (61.9%)	494 (61.8%)	142 (62.0%)	71 (61.7%)

Surgery	Yes	1015 (88.7%)	710 (88.8%)	203 (88.6%)	102 (88.7%)
	No	129 (11.3%)	90 (11.3%)	26 (11.4%)	13 (11.3%)

Grade	Low grade	230 (20.1%)	161 (20.1%)	46 (20.1%)	23 (20.0%)
	High grade	914 (79.9%)	641 (80.1%)	183 (79.9%)	90 (78.3%)

Location	Axial	163 (14.2%)	113 (14.1%)	33 (14.4%)	17 (14.8%)
	Extremity	774 (67.7%)	542 (67.8%)	155 (67.7%)	77 (67.0%)
	Other	207 (18.1%)	145 (18.1%)	41 (17.9%)	21 (18.3%)

**Table 3:** Evaluation metrics of different models in MRI data sets.

Model	Recall	Precision	F1	IOU	DSC	Params (M)	FLOPS (G)
MSRN	0.945	0.893	0.918	0.853	0.834	14.27	1461.23
MSFCN	0.936	0.881	0.906	0.841	0.874	20.38	1524.34
FPN	0.924	0.914	0.921	0.854	0.883	88.63	141.45
SepUNet	0.932	0.927	0.928	0.867	0.895	20.32	199.26
U-Net	0.924	0.922	0.924	0.859	0.893	17.26	160.16
Our	0.926	0.932	0.929	0.867	0.929	18.60	280.22

## Data Availability

The data used to support the findings of this study are currently under embargo while the research findings are commercialized. Requests for data, 12 months after publication of this article, will be considered by the corresponding author.
